# Barrier membranes for tissue regeneration in dentistry

**DOI:** 10.1080/26415275.2021.1925556

**Published:** 2021-05-20

**Authors:** Jun-Ichi Sasaki, Gabriela L. Abe, Aonan Li, Pasiree Thongthai, Ririko Tsuboi, Tomoki Kohno, Satoshi Imazato

**Affiliations:** a Department of Biomaterials Science, Osaka University Graduate School of Dentistry, Suita, Japan; b Department of Advanced Functional Materials Science, Osaka University Graduate School of Dentistry, Suita, Japan

**Keywords:** Barrier membrane, GTR, GBR, dental materials, biodegradability

## Abstract

**Background: **In dentistry, barrier membranes are used for guided tissue regeneration (GTR) and guided bone regeneration (GBR). Various membranes are commercially available and extensive research and development of novel membranes have been conducted. In general, membranes are required to provide barrier function, biosafety, biocompatibility and appropriate mechanical properties. In addition, membranes are expected to be bioactive to promote tissue regeneration.

**Objectives:** This review aims to organize the fundamental characteristics of the barrier membranes that are available and studied for dentistry, based on their components.

**Results: **The principal components of barrier membranes are divided into nonbiodegradable and biodegradable materials.

Nonbiodegradable membranes are manufactured from synthetic polymers, metals or composites of these materials. The first reported barrier membrane was made from expanded polytetrafluoroethylene (e-PTFE). Titanium has also been applied for dental regenerative therapy and shows favorable barrier function. Biodegradable membranes are mainly made from natural and synthetic polymers. Collagens are popular materials that are processed for clinical use by cross-linking. Aliphatic polyesters and their copolymers have been relatively recently introduced into GTR and GBR treatments. In addition, to improve the tissue regenerative function and mechanical strength of biodegradable membranes, inorganic materials such as calcium phosphate and bioactive glass have been incorporated at the research stage.

**Conclusions:** Currently, there are still insufficient guidelines for barrier membrane choice in GTR and GBR, therefore dentists are required to understand the characteristics of barrier membranes.

## 1. Introduction

In dentistry, barrier membranes are used to improve the prognosis in the regeneration of periodontal tissue, including in the bifurcation area and bone augmentation associated with implant treatment [1–3]. In 1982, Nyman et al. [4] succeeded in forming new attachment to tooth in human by directing periodontal tissue regeneration using a barrier membrane. Studies of dental regeneration have since advanced and operation protocols such as guided tissue regeneration (GTR) and guided bone regeneration (GBR) have been widely accepted for clinical application [5,6]. Barrier membranes implanted over the tissue defect area prohibit cell invasion from the gingival epithelium and connective tissue [7,8]. It has been reported that the shielding function is required to last 4–6 weeks for periodontal tissue regeneration and 16–24 weeks for bone augmentation [9,10], therefore barrier membranes need to persist between the gingiva and alveolar bone for longer than these time frames. This shielding function maintains the space for tissue regeneration and selectively guides the periodontal ligament derived cells or bone formation cells to the defect area [11,12].

To date, several clinical outcomes have been reported using various barrier membranes [13–15]. The required properties of barrier membranes are high biocompatibility, low permeability to cells, tight adhesion to host tissues, moderate mechanical strength, storage stability and handleability for clinical use [16,17]. In terms of the mechanical properties, the barrier membrane should be able to withstand the pressure of overhanging gingiva and keep its shape to maintain the regenerative space [18]. In addition, membranes should easily deform plastically without fracturing and maintain their morphology after implantation.

Barrier membranes are designed to promote tissue regeneration and can be divided according to the biodegradability of the base material. In recent years, the use of biodegradable membranes has been mainstreamed in GBR, however nonbiodegradable products are often applied to massive tissue loss and vertical bone defects owing to their advantages in space making [19–21]. The field of barrier membranes is expanding, and the evolution of biomaterials is inevitable. Consequently, selecting a membrane for clinical application will involve more than the current considerations of biodegradability, it will be necessary to understand other membrane components. Furthermore, even for currently commercially available membranes, guidelines for prescription protocols are not defined further than the biodegradability aspect, leaving the material selection highly dependent on the professional experience of clinician. This review aims to summarize the fundamental characteristics of the barrier membranes commercially available and currently studied in dentistry–based on their components–and provide an update from the material point of view. A better understanding of the available barrier membranes will lead to a better selection for each clinical situation.

## 2. Search methodology

A search at PubMed/MEDLINE and Scopus was performed for documents published in English using the following keywords: guided bone regeneration, guided tissue regeneration, bone augmentation, barrier membranes, functionally graded membranes, dental materials, biomaterials and biodegradability. Documents published within the past 20 years were selected and we further screened the bibliographies of the selected articles to identify relevant classical or groundbreaking studies. We discuss aspects of significant clinical impact, and opinions expressed here are also based on our own research and acquired knowledge.

## 3. Nonbiodegradable membranes

Barrier membranes composed of nonbiodegradable material is commonly used for relatively large-scale tissue regeneration owing to the ease of control of the shielding period (Table 1) [22]. Furthermore, they have the advantage that degradation by-products of the base materials do not need to be considered [23,24]. However, these membranes require surgical removal after tissue regeneration. It has been reported that nonbiodegradable membranes showed higher risk of complications related to membrane exposure during implantation than biodegradable membranes [25–27]. They can also be used in combination with metal pins and mini screws to avoid the collapse of their morphology [28,29].

**Table 1.** Summary of nonbiodegradable barrier membranes.

Materials	Advantages	Disadvantages	References
Polymers			
Polytetrafluoroethylene (PTFE), expanded PTFE (e-PTFE), dense PTFE (d-PTFE), titanium-reinforced PTFE	- High chemical stability - High biocompatibility - High barrier function	- Surgical removal required - Membrane exposure	[25, 26, 30, 32, 34–38]
Metals			
Titanium, titanium alloy	- High biocompatibility - High barrier function - Mechanical strength, durability	- Surgical removal required - Expensive	[51–55]
Cobalt, cobalt alloy	- Low cost - High mechanical strength - Solid space-making	- Less biocompatibility	[56, 57]

### 3.1. Nonbiodegradable synthetic polymers

Polytetrafluoroethylene (PTFE) is an example of a material used in nonbiodegradable membranes. The first reported barrier membrane was made from expanded PTFE (e-PTFE) [4,30–32]. PTFE is a stable polymer *in vivo* and it is categorized as a bioinert material [33,34]. This chemical stability, which counts in favor of biocompatibility, allows PTFE to endure biodegradation and prevents host immune responses. PTFE has high barrier function between tissues, therefore tends to reduce the blood supply resulting in dehiscence of the gingiva [35,36].

PTFE only membranes can be used for treatment, however titanium-reinforced membranes are common owing to their effective space-making. Recently, dense PTFE (d-PTFE), a compact form of PTFE, has been launched onto the market, and its efficacy for GBR has been evaluated [37]. Ronda et al. [38] reported that d- and e-PTFE membranes showed identical clinical results in the treatment of vertical bone defects. However, it was indicated that surgical removal was easier for the d-PTFE membrane than for the e-PTFE analogs, which could represent less disturbance to the regenerated tissue below.

### 3.2. Metals

Titanium—a popular material in dentistry as well as other medical fields—is used as both a pure metal and an alloy containing non-precious metals (e.g. aluminum, vanadium or nickel) [39–42]. Both the pure metal and alloys possess good biocompatibility, mechanical strength, durability, low density and corrosion resistance [43,44]. In addition, titanium is a bioinert material that can be used as a stable metal owing to the rapid formation of a passive layer [45,46]. Titanium is primarily used for bone fixation after maxillofacial surgery and has shown favorable outcomes [47]. As a result, titanium has been used for dental implants owing to its effective osseointegration showing direct joint to the bone through extracellular matrices [48–50]. Titanium membranes are more expensive than other membranes; however, they provide effective shape and can be applied for vertical and severe horizontal bone loss in combination with bone substitutes [51–55]. Recently, Hasegawa et al. [55] designed regular hexagonal honeycomb structure with 1 mm of inner circle in the titanium membrane. The authors also arranged microperforations with a diameter of 20 μm at 50-μm intervals within each honeycomb section. They implanted autologous bone to the bony defects created in the Beagle dogs and covered with the prototype membrane. In this study, it was demonstrated that mature osseous tissue was formed after 26 weeks of implantation. However, microperforations might be a difficulty when retrieving the membrane at the second surgery. Epithelial and connective tissue that invades the perforations will inevitably be removed with the membrane, potentially causing discomfort to the patient, and prolonging the healing period.

The use of cobalt and cobalt-chromium alloy in barrier membranes has been reported [56,57]. Decco et al. [56] applied a cobalt-chromium membrane to a noncritical bone defect in rabbit tibia and reported that the tested membrane showed solid space-making and favorable bone augmentation. Cobalt-chromium alloy is a bioinert metal like titanium, but wear processes can lead to the release of toxic chromium and cobalt ions into the body [58]. Cobalt-chromium alloy has the advantages of mechanical strength and low cost compared with titanium however it shows poorer biocompatibility than titanium, therefore there have been no clinical trials using cobalt-chromium membrane implants in humans.

The reliable space-making ability of nondegradable membranes is because of their high mechanical strength, however changes in the metal composition do not seem to provide a clear advantage of one composition over another. On the other hand, these materials require a retrieval surgery, which can damage the regenerated tissue, prolong the healing period and increase the risk of infection.

## 4. Biodegradable membranes

Biodegradable membranes, which are almost exclusively polymer-based (natural and synthetic polymers), have the advantages of few complications and low cost, as well as secondary surgeries not being necessary (Table 2) [59–61]. Therefore, biodegradable membranes are regarded a first-choice material when the treatment outcome is expected to be the same as that using non-biodegradable materials. However, biodegradable membranes are liable to show tissue regeneration failure due to the volume loss of the membrane and its degradation by-products [62,63]. For animal-derived collagen membranes, residual virus and cross-linker also present concerns [62,64,65]. In general, biodegradable membranes show lower mechanical strength and are therefore less efficient at space-making than nonbiodegradable PTFE and titanium mesh. Therefore, in GBR, bone substitutes are used in combination with biodegradable membranes to maintain the membrane shape and lead to space-making.

**Table 2.** Summary of biodegradable barrier membranes.

Materials	Advantages	Disadvantages	References
Natural polymers			
Collagen	- High biocompatibility - Favorable barrier function - Promotion of wound healing - No surgical removal	- Hard to control biodegradation - Low mechanical strength - Residual cross-linking agents - Possible disease transmission	[62, 66, 71–73, 76–79]
Other natural polymers (alginate, chitosan)	- Favorable biocompatibility - No surgical removal	- Questionable barrier function - Hard to control biodegradation - Few studies	[80–87]
Synthetic polymers			
Aliphatic polyesters (PLA, PGA and PCL), these copolymers	- Favorable biocompatibility - Favorable barrier function - High reproducibility - Controllable biodegradability and mechanical properties - No surgical removal	- Relatively low mechanical strength - Cytotoxicity of degradation byproduct	[95–100, 102]

### 4.1. Natural polymers

**Figure 1.**
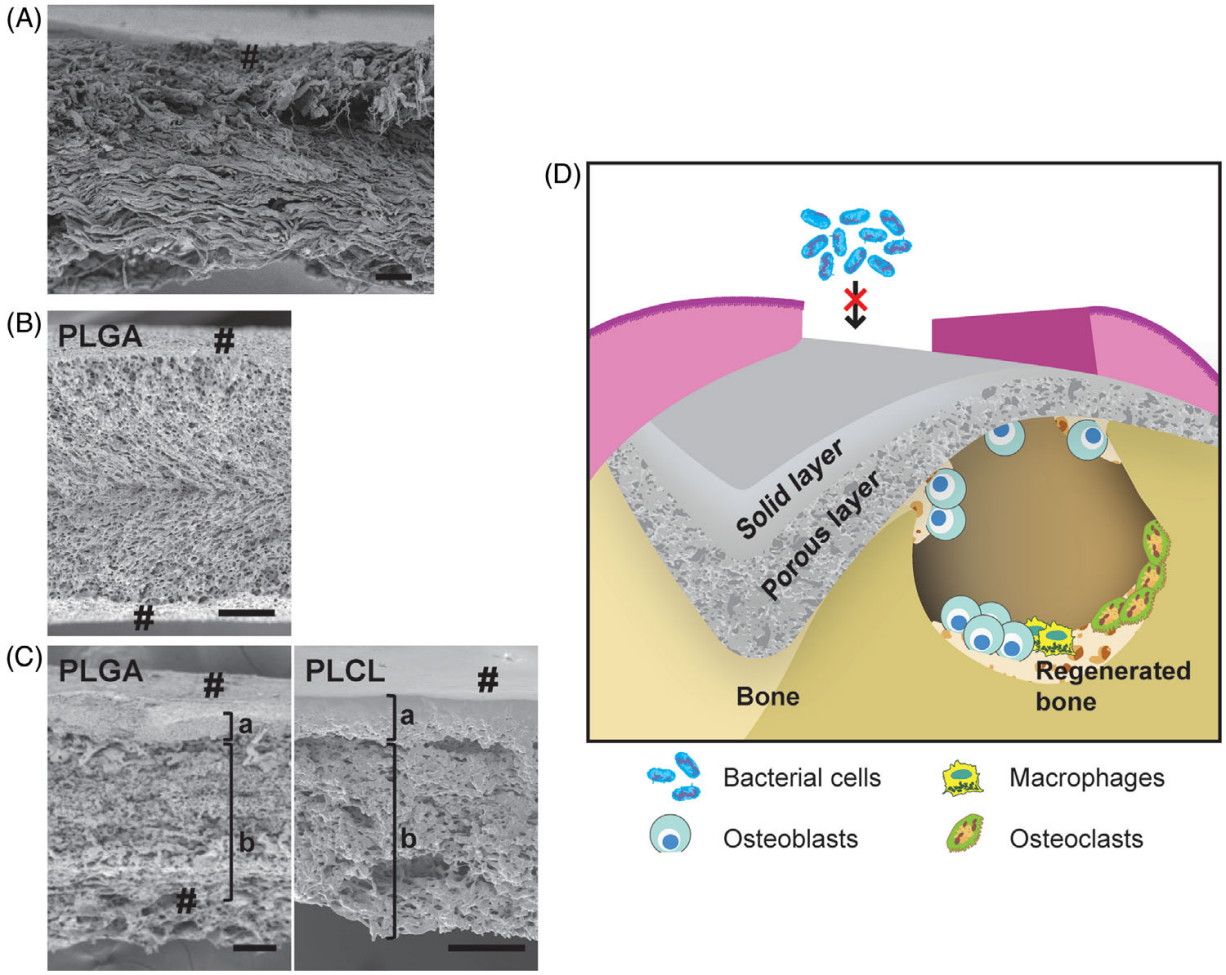
Cross-sectional images and schematic illustration of GBR membrane. Cross-sectional electron micrographs of (A) collagen membrane (Bio-Gide®), (B) PLGA monolayer membrane and (C) bilayer membranes composed of PLGA (left) and PLCL (right). (D) Schematic illustration of a bilayer membrane for GBR. #: membrane surface; a: solid layer and b: porous layer (b) (scale bar: 100 μm). Reproduced with permission from Yoshimoto et al. [97] and Abe et al. [100]. Copyright Elsevier B.V.

Type I and type III collagens derived from bovine and porcine are the commonly used materials for natural polymer membranes (Figure 1(A)) [66,67]. Collagen is an essential component of bone and connective tissue and supports these tissue structures [68–70]. Collagens are harvested from epidermis, tendon and intestine, then processed by decellularization, cross-linking treatment and sterilization to produce the barrier membranes [71]. There are various methods for cross-linking including ultraviolet irradiation and chemical treatment using glutaraldehyde, water-soluble carbodiimide and genipin [71,72]. These treatments increase hydrolysis resistance and membranes that can persist for six months within the human body have been developed for GBR treatment [9,73]. However, the residual chemical cross-linking agents were problematic for clinical usage due to their potential toxicity causing inflammation and interfering with cellular processes [74]. The cross-linking of structures improves the mechanical properties to an extent; however, the low stiffness of the membranes remains a drawback [71,75]. Therefore, collagen membranes were better suited to application in areas that are simple to set.

There are some reports of the bone augmentation efficiency of collagen membranes in GBR [76,77]. The authors showed that the bone formation with collagen membranes was comparable to the amount observed for e-PTFE membrane. It was also reported that the collagen membranes contributed to bone regeneration as well as the passive barrier [78,79]. Stromal cells attached to the collagen membrane promoted the production of basic fibroblast growth factor (FGF)-2 and bone morphogenetic protein (BMP)-2 and these growth factors facilitated bone regeneration compared with other biodegradable membranes.

Chitosan and alginate have also been introduced for use as barrier membranes in the study phase [80–87]. Chitosan—a straight-chain polysaccharide, copolymer of glucosamine and N-acetyl-D-glucosamine—is industrially produced by the alkaline treatment of crustacean chitin [88,89]. Chitosan exhibits biodegradability, favorable biocompatibility and flexible workability, therefore chitosan processed in fibrous, film and spongy forms has been applied to surgical sutures and artificial skin [90,91]. It is thought that the biodegradability of chitosan depends on its molecular weight [92]. Although chitosan-based membranes showed favorable bioactive properties, such as bacteriostatic and homeostatic abilities, it still retains low mechanical properties as other natural polymers.

Alginate, a natural polymer extracted from seaweed, is a popular impression material in dentistry. Alginate hydrogel shows high biocompatibility and has a similar structure to extracellular matrix [89,93,94]. An *in vivo* study showed that chitosan and alginate membranes can function as barrier membranes, however there are currently no clinical trials assessing their performance in humans, so they are not applied in the clinic [80–82,87]. This may be because these materials have not been proven superior to their commercial counterparts.

### 4.2. Biodegradable synthetic polymers

Biodegradable aliphatic polyesters—such as polylactic acid (PLA), polyglycolic acid (PGA), polycaprolactone (PCL) and their copolymers (e.g. poly(lactic-co-glycolic acid) (PLGA) and poly(lactide-co-caprolactone) (PLCL))—are also used as barrier membranes [67,95–100]. Aliphatic polyester membranes can be made to be reproducible because these workable materials are processed industrially. The advantages of aliphatic polyester membranes include their adjustable biodegradability and mechanical properties, which can be controlled by regulating the polymer composition [101,102]. In addition, therapeutics and materials that promote tissue regeneration can be easily impregnated into the membranes [103]. The biodegradability of aliphatic copolymer varies significantly depending on the types and ratio of polyester. The addition of PCL, which shows greater hydrophobicity and lower degradability than PLA and PGA, allows the lifetime of the membrane to be extended [100]. However, improving the mechanical properties using only biodegradable polyester is challenging, therefore these membranes tend to be applied for small tissue defects or in combination with bone substitute.

Recently, our group developed bilayer membranes based on PLGA or PLCL [97,100]. These membranes comprised a solid layer and a porous layer, which respectively provided barrier function and cell support (Figure 1(B,C)). The thickness of each layer could be regulated by adjusting the temperature used for freeze drying. These membranes showed lower mechanical strength but better operability than the monolayer membranes. It was shown that the porous structure promoted cell proliferation and osteogenic differentiation of mesenchymal stem cells. It was also shown that the PLGA bilayer membrane was able to promote bone regeneration *in vivo*, where bone formation with the PLGA bilayer was significantly higher than that with a monolayer membrane [97]. As regards PLCL bilayer membrane, we have demonstrated that solid layer reduced bacterial adhesion and prevented bacterial invasion inside the membrane [104]. This characteristic could improve the prognosis and simplify the management of GTR/GBR complications. Furthermore, the PLCL membrane has a slower degradation rate than the other biodegradable polymers. Thus, PLCL bilayer membranes are considered promising biomaterials for GBR treatment (Figure 1(D)) [100].

No polymer, natural or synthetic, seem to be sufficient on its own. The association of different materials has the potential to combine their best features. Bioactivity and mechanical strength are the main target of the current attempts to improve polymeric membranes, and it is in this context that the incorporation of additives is proposed.

## 5. Membrane additives

There are some reports that incorporating inorganic components into barrier membranes promotes bone regeneration and improves the mechanical strength [86,105–108]. Hydroxyapatite, a calcium phosphate, is widely used in bone regenerative medicine and shows nonbiodegradability and osteoconductive properties [109,110]. Some research groups have attempted to add hydroxyapatite to barrier membranes for GTR and GBR applications [86,106,107,111]. Basile et al. [107] combined nanowhisker hydroxyapatites and modified PCL and reported that the composite membrane showed favorable proliferation and osteogenic differentiation of mesenchymal stromal cells *in vitro*. Veríssimo et al. [106] fabricated hydroxyapatite deposited collagen membranes and implanted them into critical-sized calvaria defects. The authors reported that the experimental membranes accelerated bone healing, but lost their biodegradability.

Beta-tricalcium phosphate (β-TCP)—categorized as a calcium phosphate material, similarly to hydroxyapatite—also possesses osteoconductivity and biodegradability [111–113]. Shim et al. [105] blended PLGA, PCL and β-TCP and fabricated a mesh membrane for GBR. The authors reported that this composite membrane promoted proliferation and osteogenic differentiation of mesenchymal stem cells *in vitro*, and increased bone formation without using bone substitute *in vivo*.

Bioactive glass composed mainly of silicon dioxide is an amorphous material that shows biodegradability [114–116]. Bioactive glass can release calcium and silicate ions, enhancing the activity of osteoblasts, and as a result forms a connection with bone [116–118]. It has been shown that bioactive glass contained in biodegradable membranes promoted mineral deposition on the surface and osteoblastic cell functions [84,87,98,119,120]. Hong et al. [119] combined a bioactive glass with collagen membrane, to which FGF-2 solution was infiltrated. The authors implanted the hybrid membranes in rat calvaria defects, which subsequently showed accelerated bone regeneration.

## 6. Conclusion

As a result of the progress in polymer science, barrier membranes have become widely used in dentistry. The membranes for GTR and GBR are expected to possess multiple properties that if effectively combined would develop an excellent material. More recently, bioactive functions of membrane have been experimented with, aiming at the enhanced promotion of tissue regeneration. To achieve these high standards, future research must explore the combination of growth factors and antibacterial agents in barrier membranes, as well as the dynamics of material degradation.

There are still no guidelines for choosing barrier membranes for GTR and GBR apart from the general considerations of selecting biodegradable versus nonbiodegradable membrane. Consequently, the protocols and materials used for these treatments depend greatly on the shortcomings of each membrane, and on the professional experience and skill of the dentists. Therefore, dentists must understand the characteristics of barrier membranes, as well as bone substitutes and growth factors, to select a suitable barrier membrane.
